# 

**Published:** 1850-01

**Authors:** 


*New Publications.*—Several new medical works have been received, which will be noticed in our next number.

